# Biological Activities of α-Pinene and β-Pinene Enantiomers

**DOI:** 10.3390/molecules17066305

**Published:** 2012-05-25

**Authors:** Ana Cristina da Silva Rivas, Paula Monteiro Lopes, Mariana Maria de Azevedo Barros, Danielle Cristina Costa Machado, Celuta Sales Alviano, Daniela Sales Alviano

**Affiliations:** 1 Institute of Microbiology Paulo de Góes, Federal University of Rio de Janeiro (IMPG-UFRJ), CCS, Ilha do Fundão, Rio de Janeiro, RJ 21941-590, Brazil; 2 Chemistry Institute, Federal University of Rio de Janeiro (UFRJ), CT, Ilha do Fundão, Rio de Janeiro, RJ 21941-909, Brazil

**Keywords:** pinenes, isomers, enantiomers, antimicrobial activity

## Abstract

The antimicrobial activities of the isomers and enantiomers of pinene were evaluated against bacterial and fungal cells. The agar diffusion test showed that only the positive enantiomers of the α- and β-isomers of pinene were active. The minimal inhibitory concentration (MIC) and minimal microbicidal concentration (MMC) of these monoterpenes were also determined, confirming that the positive enantiomers exhibited microbicidal activity against all fungi and bacteria tested with MICs ranging from 117 to 4,150 µg/mL. However, no antimicrobial activity was detected with the negative enantiomers up to 20 mg/mL. Time-kill curves showed that (+)-α-pinene and (+)-β-pinene were highly toxic to *Candida albicans*, killing 100% of inoculum within 60 min. By contrast, the bactericidal effect occurred after 6 h in methicillin-resistant *Staphylococcus aureus* (MRSA). In combination with commercial antimicrobials, ciprofloxacin plus (+)-α-pinene or (+)-β-pinene presented synergistic activity against MRSA whereas an indifferent effect against all fungi was detected when amphotericin B was combined with the positive enantiomers of pinene. The potential of (+)-α-pinene and (+)-β-pinene to inhibit phospholipase and esterase activities was also evaluated, and the best inhibition results were obtained with *Cryptococcusneoformans*. *C. albicans* biofilm formation was prevented with the MIC concentration of (+)-α-pinene and twice the MIC value of (+)-β-pinene. Finally, the cytotoxicity of the positive enantiomers of pinene to murine macrophages was evaluated, and 250 µg/mL of (+)-α-pinene and (+)-β-pinene reduced the cell viability to 66.8% and 57.7%, respectively.

## 1. Introduction

Several essential oils have been used as therapeutic agents since ancient times, and some of them have been scientifically proven to possess medicinal properties, including anti-inflammatory [1], antiviral [2], antitumor [3], cytotoxic [4], and antimicrobial activities [5]. Essential oils are complex mixtures of volatile, lipophilic and odiferous substances from the secondary metabolism of plants. They are mainly composed of monoterpenes, sesquiterpenes and their oxygenated derivatives (alcohols, aldehydes, esters, ketones, phenols and oxides).

Many substances within living organisms are chiral and can occur alone (only one enantiomer) or in racemic mixtures, with the same or distinct functions. For example, carvone is used as a essence for perfumes, and each enantiomer has a different odor: *S*(+)-carvone smells like spearmint and *R*(−) smells like caraway [6]. Linalool enantiomers show the same antimicrobial activity against several microorganisms, especially against the fungus *Botrytis cinerea* and the protozoan *Plasmodium falciparum* [7]*.* Goniothalamin enantiomers similarly inhibit antifungal growth and biofilm progression against *Candida* species [8].

Pinenes and bicyclic terpenes can be found in the essential oils of coniferous trees (pine), rosemary, lavender, and turpentine. These compounds exist as optical isomers or enantiomers that do not overlap with each other’s mirror images and they differ only in their interaction with polarized light [9]. These compounds may exhibit differences in toxicity and biological activity [10,11].

**Figure 1:**
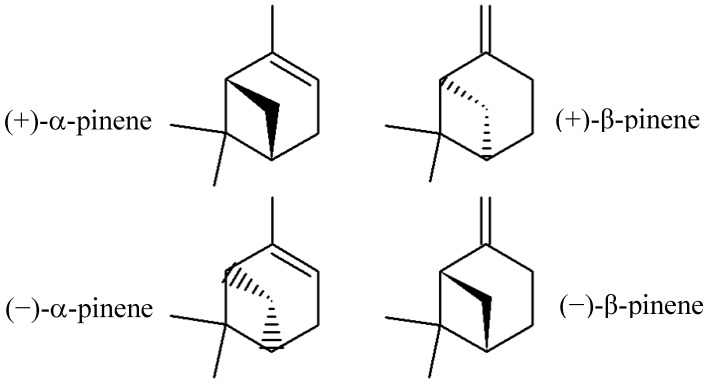
Structural formulas of α-pinene and β-pinene enantiomers.

Pinenes have two active constitutional isomers: α- and β-pinene. Both structural isomers have enantiomers known in nature as (−)-α-pinene (more common in European pines), (+)-α-pinene (more common in North America), (−)-β-pinene and (+)-β-pinene. The racemic mixture is present in some essential oils, such as eucalyptus oil [9,10,11]. Figure 1 shows the structural formulas of α-pinene and β-pinene enantiomers.

In plants, pinenes show fungicidal activity and have been used for centuries to produce flavors and fragrances. Several biological activities are associated with pinenes, including use as a natural insecticide. The negative enantiomers exhibit antiviral effects against infectious bronchitis virus (IBV) [11,12]. However, there is no consensus regarding the antimicrobial activity of pinenes, potentially because of the lack of enantiomer identification. Some authors have attributed the antimicrobial activity of some essential oils to these monoterpenes [13,14,15]. On the other hand, others have reported that pinenes exhibit no antimicrobial activity [10,16,17]. To understand the controversial results concerning the antimicrobial activity of pinenes, this work aimed to evaluate the antimicrobial effects of the different isomers and enantiomers of these monoterpenes against *Candida albicans*, *Cryptococcus neoformans*, *Rhizopus oryzae* and methicillin-resistant *Staphylococcus aureus* (MRSA).

## 2. Results and Discussion

### 2.1. Minimal Inhibitory Concentration (MIC) of (+)-*α*-Pinene and (+)-β-Pinene Standards

The MIC values of α-pinene and β-pinene enantiomers were determined. Only the positive enantiomers exhibited a microbicidal effect against all of the microorganisms tested, with MIC values ranging from 117 µg to 6,250 µg/mL. No antimicrobial activity was detected with the negative enantiomers up to 20 mg/mL. Fungi, especially *C. neoformans*, were more sensitive to (+)-α-pinene and (+)-β-pinene than MRSA (Table 1).

**Table 1:** Minimal inhibitory concentrations (MICs) of the isomers and enantiomers of pinene and antimicrobial drugs.

Microorganisms	MIC * (µg/mL)					
	(+)-α-pinene	(−)-α-pinene	(+)-β-pinene	(−)-β-pinene	AMB	CIP
*C. albicans*	3,125	na	187	na	0.125	-
*C. neoformans*	117	na	234	na	0.125	-
*R. oryzae*	390	na	780	na	0.48	-
MRSA	4,150	na	6,250	na	-	0.5

* All MICs were microbicidal; na—no activity; AMB—amphotericin B; CIP—ciprofloxacin.

### 2.2. Time-Kill Curves

**Figure 2:**
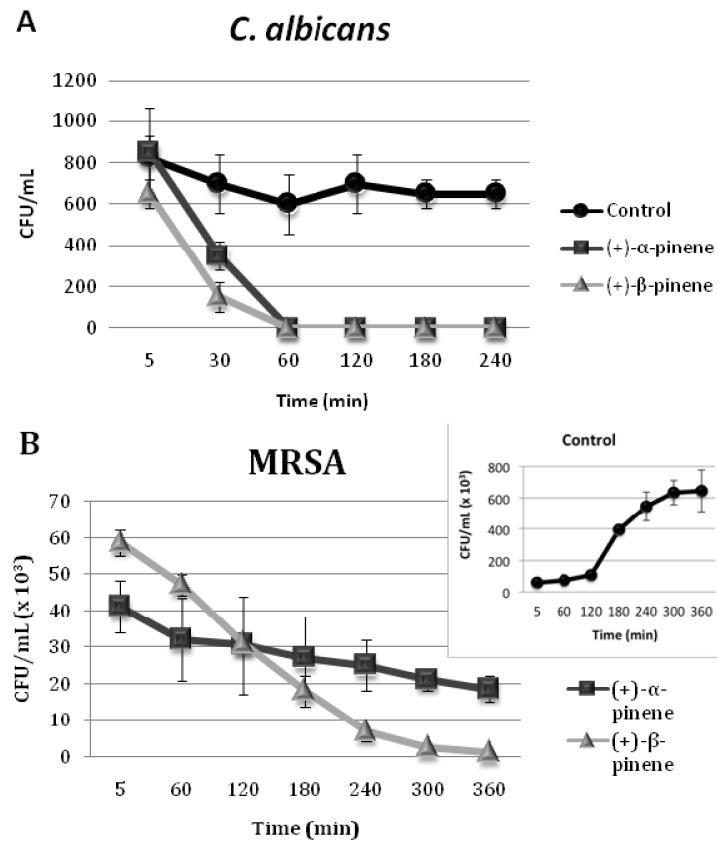
Time-kill curves of *C. albicans* (**A**) and MRSA (**B**) treated with (+)-α-pinene and (+)-β-pinene.

Because all of the (+)-α-pinene and (+)-β-pinene MICs were microbicidal, time-kill curves were determined for *C. albicans* and MRSA. The killing time of both microorganisms maintained in the presence of MICs of (+)-α-pinene and (+)-β-pinene was determined. The positive enantiomers were able to eliminate 100% of *C. albicans* in 60 min (Figure 2A). However, total killing of MRSA only occurred after 6 h of incubation (Figure 2B). Another study showed that terpenes such as citral and linalool were able to eliminate 100% of *C. albicans* ATCC 10231 in 60 min, whereas eugenol and citronellal took 120 min [18].

### 2.3. Synergistic Activity of (+)-*α*-Pinene and (+)-β-Pinene Standards with Commercial Antimicrobials against Microorganisms

Synergistic activities were measured with the checkerboard assay, which was conducted to evaluate the antimicrobial effect of combinations of antimicrobial drugs and pinene standards [18]. Amphotericin B and CIP were combined with (+)-α-pinene and (+)-β-pinene standards. All combinations produced FIC_index_ values ranging from 0.256 to 1.93, corresponding to synergistic or indifferent effects (Table 2). All of the combinations reduced the MIC values of at least one of the paired substances. However, the synergistic effect only occurred when CIP was combined with (+)-α-pinene or (+)-β-pinene, reducing MIC values from 4,150 to 1,037 µg/mL of (+)-α-pinene and from 6,250 to 662 µg/mL of (+)-β-pinene when they were combined with 0.003 (166-fold reduction in MIC) and 0.06 μg/mL (8-fold reduction in MIC) of CIP, respectively. There are few studies on the synergism of antimicrobial drugs and terpenes. Zore and colleagues demonstrated that terpenes such as eugenol, citronellal, citral, and linalool combined with fluconazole caused synergistic effects against strains of *C. albicans* ATCC 10231 [18].

**Table 2:** Susceptibilities of microorganisms to antimicrobial drugs in combination with (+)-α-pinene and (+)-β-pinene standards (µg/mL).

M.o.	(+)-α-pinene and AMB			(+)-α-Pinene and CIP			(+)-β-Pinene and AMB			(+)-β-Pinene and CIP		
	MIC in combination		FIC index	MIC in combination		FIC index	MIC in combination		FIC index	MIC in combination		FIC index
	α-pinene	AMB		α-pinene	CIP		β-pinene	AMB		β-pinene	CIP	
MRSA	-	-	-	1037	0.003	0.256 (S)	-	-	-	662	0.06	0.226 (S)
*C. albicans*	390	0.06	0.62 (I)	-	-	-	187	0.11	1.93 (I)	-	-	-
*C. neoformans*	78.12	0.053	1.10 (I)	-	-	-	78.12	0.11	1.29 (I)	-	-	-
*R. oryzae*	390	0.026	1.05 (I)	-	-	-	390	0.11	0.72 (I)	-	-	-

M.o.—microorganisms; AMB—amphotericin B; CIP—ciprofloxacin; S—synergistic; I—indifferent; FIC index—fractional inhibitory concentration index.

### 2.4. Inhibition of Microbial Phospholipase and Esterase Activities

**Figure 3:**
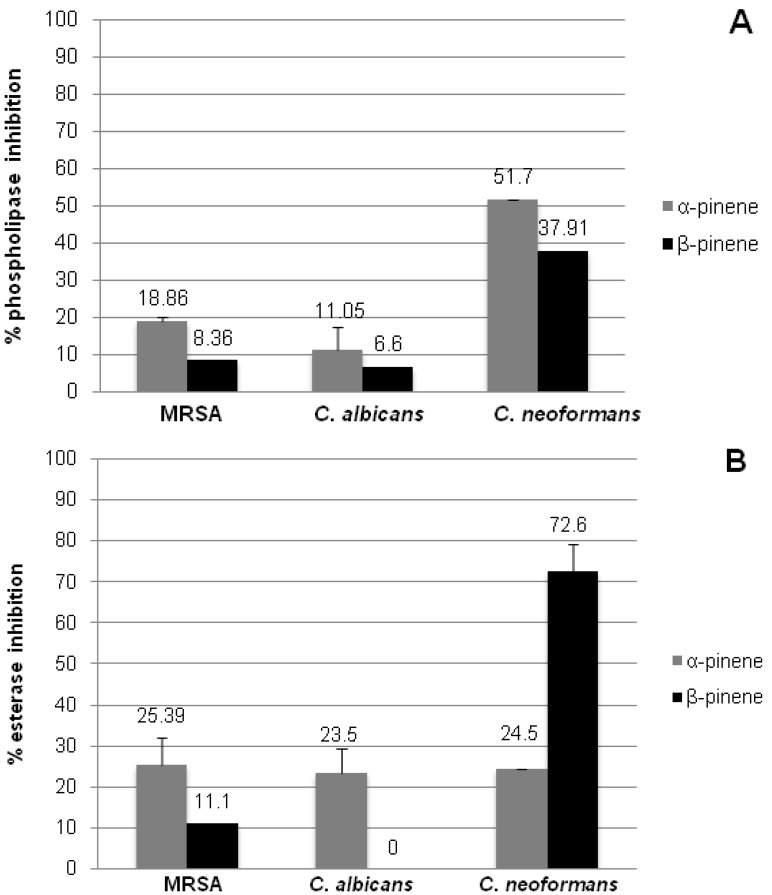
Effect of (+)-α-pinene and (+)-β-pinene standards on the inhibition of phospholipase (**A**) and esterase (**B**) activities secreted by *C. albicans*, *C. neoformans* and MRSA treated with subinhibitory (sub-MIC) concentrations. The results represent the mean ± standard error of two independent experiments in triplicate.Values over the bars refer to the percentage of inhibition of enzymatic activity.

Media containing substrates for phospholipases and esterases were used to evaluate the inhibition of these enzymes secreted by microorganisms treated with subinhibitory concentrations (sub-MIC) of (+)-α-pinene and (+)-β-pinene standards. A decrease in hydrolysis of the substrates was very low for both MRSA and *C. albicans*. However, significant results were obtained with (+)-α-pinene sub-MIC, which inhibited 50% of the phospholipase activity, and (+)-β-pinene sub-MIC, which inhibited 72% esterase activity of *C. neoformans* (Figure 3).

### 2.5. *In vitro* Biofilm Susceptibility Assay

**Figure 4:**
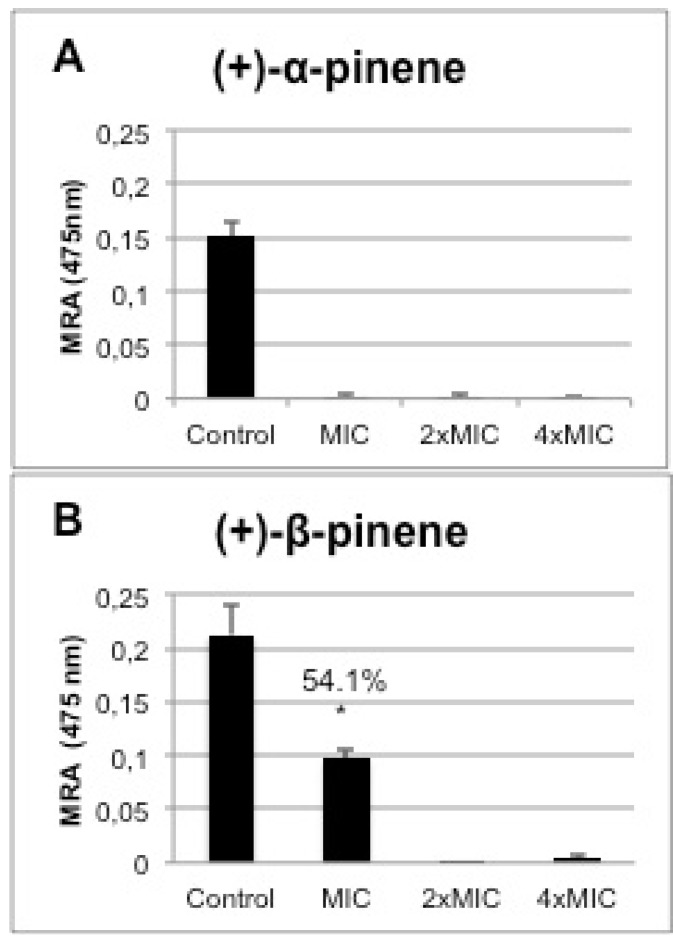
Effect of (+)-α-pinene (**A**) and (+)-β-pinene (**B**) standards on the mitochondrial reducing activity (MRA) of biofilms formed by *C. albicans* ATCC 10231 treated with one-,two- and four-fold MIC concentrations. The results represent the mean ± standard error of two independent experiments in triplicate. Values over the bars refer to the percentage of inhibition of biofilm viability; * *p* < 0.001.

*C.albicans* ATCC10231 biofilm formation in the presence of one-, two- and fourfold MIC of (+)-α-pinene and (+)-β-pinene was reduced significantly when compared with control biofilm formed in the absence of the substances. Biofilm formation was 100% inhibited by the MIC of (+)-α-pinene (Figure 4A). Although twofold MIC of (+)-β-pinene prevented biofilm formation, the MIC significantly reduced it by 54% (*p*< 0.01) (Figure 4B). Biofilms are well-structured communities of microorganisms that are extremely resistant to antibiotics [19]. Mowat and colleagues observed that susceptibility to antifungal agents such as itraconazole, fluconazole, and amphotericin B was a thousand times lower in biofilms compared with planktonic cells of *Aspergillus fumigatus* [20].

### 2.6. Cytotoxicity of the Positive Enantiomers of Pinene

**Figure 5:**
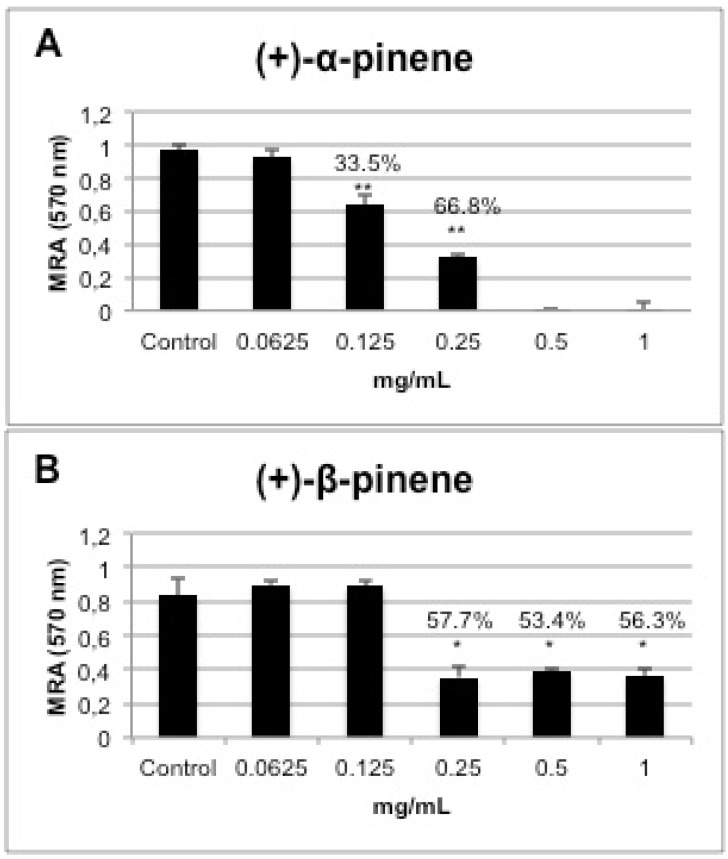
Effect of (+)-α-pinene (**A**) and (+)-β-pinene (**B**) standards on mitochondrial reducing activity (MRA) of Swiss mouse peritoneal macrophages treated with 0.0624 to 1 mg/mL of pinene. The results represent the mean ± standard error of two independent experiments in triplicate. Values over the bars refer to the percentage of inhibition of viability; * *p* < 0.01, ** *p* < 0.001.

The cytotoxic effect of (+)-α-pinene and (+)-β-pinene standards against murine macrophages was evaluated at concentrations ranging from 62.5 µg/mL to 1 mg/mL. The results shown in Figure 5 indicate the percentage of inhibition of the mitochondrial activity of Swiss mouse peritoneal macrophages determined using the XTT technique. A comparison of both enantiomers revealed that (+)-α-pinene was more cytotoxic, reducing cell viability by 33.5% with 0.125 mg/mL and by 100% with 0.5 mg/mL (Figure 5A). Despite being cytotoxic, (+)-β-pinene reduced macrophage viability by 57% at concentrations of 0.25, 0.5 and 1.0 mg/mL, and was not toxic at 0.125 mg/mL (Figure 5B).

## 3. Experimental

### 3.1. Chemicals and Microorganisms

Amphotericin B (AMB), ciprofloxacin (CIP) and standards of (+)-α-pinene (≥99%), (−)-α-pinene (98%), (+)-β-pinene (≥98.5%) and (−)-β-pinene (97%) were obtained from Sigma-Aldrich (Brazil) and stored according to the supplier’s instructions. *Candida albicans* ATCC 10231, *Cryptococcus neoformans* T_1_-444 Serotype A (Universidade Federal de São Paulo, UNIFESP), and *Rhizopus oryzae* UCP1506 (Universidade Católica de Pernambuco) were maintained in Sabouraud agar, and *Staphylococcus aureus* MRSA BMB9393 (Hospital Clementino Fraga Filho—UFRJ) was kept in brain heart infusion (BHI) agar.

### 3.2. Minimal Inhibitory Concentration (MIC) of Pinene Standards against Microorganisms

The MICs of (+)-α-pinene, (−)-α-pinene, (+)-β-pinene, and (−)-β-pinene were determined using a broth microdilution test as recommended by CLSI M27-A3 for yeast, M38-A2 for filamentous fungus, M7-A4 for bacteria [21]. After two-fold serial dilution of test substances, wells were inoculated with 10 µL of the bacterial suspension in Mueller Hinton or inoculated with 100 µL of the fungal suspension in RPMI-MOPS pH 7.2. The microplates were incubated overnight at 37 °C for MRSA and for 48 h at room temperature (28–30 °C) for fungi. Pure medium was used as the negative control, and positive controls comprised inoculated growth medium. The results were based on visual growth of microorganisms, which were confirmed with 30 µL of resazurin (Sigma-Aldrich) added aseptically to the microplate wells and incubated at 37 °C for 1 h. The MIC was defined as the minimal concentration of the antimicrobial agent presenting complete growth inhibition. Amphotericin B and ciprofloxacin were used as antimicrobial standards.

### 3.3. Time-Kill Curves

Time-kill curves can be used to evaluate the minimum time required for the death of the microorganisms. The method consisted of measuring the kinetics of the antimicrobial activity of pinene standards through time-dependent curves of death [18]. *C. albicans* in RPMI-MOPS (pH 7.2) and MRSA in Mueller Hinton (10^3^ cells/mL) were maintained in the presence of MICs of (+)-α-pinene and (+)-β-pinene. Then, 50 μL aliquots were removed after 5, 30, 60, 120, 180 and 240 min, diluted in 150 μL of sterile saline and plated onto BHI agar. After 24–48 h of incubation, the colony-forming units (CFUs) were counted and compared with that of the control, which was free of pinene standards.

### 3.4. Synergistic Activity of Pinenes and Antimicrobial Drugs against Microorganisms

The effect of (+)-α-pinene and (+)-β-pinene standards on amphotericin B and ciprofloxacin synergistic antimicrobial activity was studied by a checkerboard assay with the broth microdilution method according to Zore *et al.* [18]. Commercial antimicrobials and pinenes were combined in concentrations lower than their individual MIC values by serial dilution in 96-well microtiter plates. Each plate was inoculated with 10^3^ cells/mL of microorganisms and incubated at 37 °C for 24 h. The results were based on visual growth of the microorganisms, which were confirmed with 30 µL of resazurin (Sigma-Aldrich) added aseptically to each microplate well and incubated at 37 °C for 1 h. The fluorescence intensity was measured at 560 nm (excitation) and 590 nm (emission) with a microplate reader (Spectramax M5, Molecular Devices, Sunnyvale, CA, USA). Fractional inhibitory concentrations (FICs) for each compound and in combination with commercial antimicrobials were calculated. The FIC is calculated by dividing the concentration of a compound that kills when used in combination with another compound by the concentration that has the same effect when used individually. FIC*_index_* indicates the nature of an interaction between two compounds. A FIC*_index_* between 0.5 and 4.0 indicates an insignificant interaction, whereas FIC*_index_* values <0.5 and >4.0 have synergistic and antagonistic interactions, respectively. In general, lower values of FIC*_index_* exhibit synergistic activity and higher values of FIC*_index_* show antagonistic activity.

### 3.5. Inhibition of Microbial Phospholipase and Esterase

Phospholipase inhibition was performed by using egg yolk agar plates (1 M NaCl, 5 mM CaCl_2_ and 8% sterile egg yolk emulsion) according to Price *et al.* [22]. Esterase inhibition was performed using the agar medium, which was prepared by mixing 10 g of peptone, 5 g of NaCl, 0.1 g of CaCl_2_, 15 g of agar, and 1,000 mL of distilled water, with the pH adjusted to 6.5. After the medium was autoclaved, it was cooled to about 50 °C, and 5 mL of autoclaved Tween 80 (Sigma-Aldrich) was added [23]. Suspensions of 5 × 10^2^ to 2.5 × 10^3^ cells/mL of MRSA, *C. albicans* and *C. neoformans* were treated with sub-inhibitory concentrations (half of MIC values) of (+)-α-pinene and (+)-β-pinene, and cell suspensions without treatment were used as controls. After incubation at 37 °C for 24 h, 10 µL of each suspension was placed in the center of phospholipase and esterase agar plates, which were then incubated at 37 °C up to 10 days. In both methods, substrates digested by phospholipase and esterase produced precipitation around microorganism colonies. The colony diameter (a) and the diameter of the colony plus the precipitation zone (b) were measured by a digital paquimeter and compared with the control. The Pz values (a/b) were calculated to compare the phospholipase and esterase activities [23]. High Pz values indicate low phospholipase or esterase activities. The results are presented as the percentage of inhibition of enzymatic activity compared to controls without treatment with pinenes.

### 3.6. Inhibition of Biofilm Formation

*C. albicans* ATCC 10231 was grown as a biofilm in a 96-well microtiter plate as reported previously [24]. Briefly, *C. albicans* was grown in Sabouraud agar at 37 °C overnight. After incubation, the cells were harvested and resuspended at a density of 1 × 10^7 ^ cells/mL in yeast nitrogen base broth (YNB), pH 7.0 and supplemented with 2% glucose. Microtiter plates previously coated with 100 μL of 50% fetal bovine serum (FSB) and washed with PBS were incubated with 100 µL of cell suspension for 90 min at 37 °C. Non-adherent cells were removed by washing twice with PBS, and 100 μL of different concentrations of (+)-α-pinene and (+)-β-pinene, (one-, two- and fourfold MIC) diluted in YNB medium supplemented with 2% glucose were added to the wells. The plates were incubated for up to 48 h at 35 °C under agitation. To evaluate the mitochondrial activity, the medium was removed after incubation, the wells were washed twice with PBS, and then, 150 μL of XTT-menadione (12.5 μg/mL + menadione 0.17 μg/mL) solution was added per well. After incubation at 37 °C for 2 h in the dark, 100 μL of each well was transferred to another 96-well microtiter plate, and the absorbance was measured in a microplate reader (SpectraMax M5) at a wavelength of 475 nm.

### 3.7. Cytotoxicity Assay

Swiss mouse peritoneal macrophages were maintained in RPMI-1640 medium containing 10% FBS, 1% glutamine, 1 mM sodium pyruvate, 10 mM MOPS at pH 7.4 and incubated at 37 °C in 5% CO_2_ atmosphere. In all, 1 × 10^5^ cells were inoculated in 96-well microtiter plates and incubated overnight in cell culture medium. Murine macrophages were incubated in the absence or presence of (+)-α-pinene and (+)-β-pinene at concentrations ranging from 62.5 µg/mL to 1 mg/mL for 24 h. Cell viability was determined after 24 h of treatment using 3-(4,5-dimethylthiazol-2-yl)-2,5-diphenyltetrazolium bromide (MTT). Then, 10% (v/v) of 5 mg/mL MTT was added to each well, and the plate incubated for 4 h at 37 °C and 5% CO_2_, followed by addition of DMSO and additional incubation for 1 h at room temperature. MTT was converted to dark blue, water-insoluble MTT formazan by mitochondrial dehydrogenases of living cells, allowing quantification of cell viability by measuring their metabolic function. The blue crystals were solubilized with DMSO, and the intensity was measured colorimetrically in a microplate reader (SpectraMax M5) at wavelengths of 570 nm and 655 nm [25].

### 3.8. Statistical Analysis

All of the experiments were repeated at least two times, and all of the systems were tested in triplicate. The data were analyzed statistically using Student’s *t* tests. *p* values of 0.05 or less were considered statistically significant.

## 4. Conclusions

This study showed that only the positive enantiomers of pinene have antimicrobial activity against *C. albicans*, *C. neoformans*, *R. oryzae* and MRSA. The additive and synergistic effects of (+)-α-pinene and (+)-β-pinene standards combined with commercial antimicrobials are important as they reduced the MIC of combined substances, maintained the antimicrobial activity and decreased toxicity. The significant inhibition of *C. neoformans* phospholipase and esterase activities by the pinene positive enantiomers could be related to the potent antimicrobial action of pinene against this fungus. The antimicrobial activity was even more promising against biofilm formation, which makes pinene useful in formulating strategies to limit *C.albicans* biofilm formation.

## Supplementary Materials

Supplementary File 1

## References

[1] Koudou J., Abena A.A., Ngaissona P., Bessière J.M. (2005). Chemical composition and pharmacological activity of essential oil of *Canarium schweinfurthii*. Fitoterapia.

[2] Loizzoa M.R., Saabb A., Tundisa R., Stattia G.A., Lamprontic I., Menichinia F., Gambarid R., Cinatle J., Doer H.W. (2008). Phytochemical analysis and *in vitro* evaluation of the biological activity against herpes simplex virus type 1 (HSV-1) of *Cedrus libani* A. Rich. Phytomedicine.

[3] M’Barek L.A., Ait Mouse H., Jaâfari A., Aboufatima R., Benharref A., Kamal M., Bénard J., El Abbadi N., Bensalah M., Gamouh A. (2007). Cytotoxic effect of essential oil of thyme (*Thymus broussonettii*) on the IGR-OV1 tumor cells resistant to chemotherapy. Braz. J. Med. Biol. Res..

[4] Zarai Z., Kadri A., Chobba I.B., Mansour R.B., Bekir A., Mejdoub H., Gharsallah N. (2011). The *in-vitro* evaluation of antibacterial, antifungal and cytotoxic properties of *Marrubium vulgare* L. essential oil grown in Tunisia. Lipids Health Dis..

[5] Alviano D.S., Alviano C.S. (2009). Plant extracts: Search for new alternatives to treat microbial diseases. Curr. Pharm. Biotechnol..

[6] Paiva A.P.O. (2006). Fenómeno da quiralidade: Base da Esterioquímica. Quimica.

[7] Özek T., Tabanca N., Demirci F., Wedge D.E., Baser K.H.C. (2010). Enantiomeric distribution of some linalool containing essential oils and their biological activities. Rec. Nat. Prod..

[8] Martins C.V.B., de Resende M.A., da Silva D.L., Magalhães T.F.F., Modolo L.V., Pilli R.A., de Fátima A. (2009). *In vitro* studies of anticandidal activity of goniothalamin enantiomers. J. Appl. Microbiol..

[9] Solomons T.W.G., Fryhle C.B. (2009). Química Organica.

[10] Tabanca N., Demirci B., Crockett S.L., Baser K.H.C., Wedge D.E. (2007). Chemical composition and antifungal activity of *Arnica longifolia*, *Aster hesperius*, and *Chrysothamnus nauseosus* essential oils. J. Agric. Food Chem..

[11] Yang Z., Wu N., Zu Y., Fu Y. (2011). Comparative anti-infectious bronchitis virus (IBV) activity of (−)-pinene: Effect on nucleocapsid (N) protein. Molecules.

[12] Albuquerque M.R.J.R., Costa S.M.O., Bandeira P.N., Santiago G.M.P., Andrade-Neto M., Silveira E.R., Pessoa O.D.L. (2007). Nematicidal and larvicidal activities of the essential oils from aerial parts of *Pectis oligocephala* and *Pectis apodocephala* Baker. Ann. Braz. Acad. Sci..

[13] Aligiannis N., Kalpoutzakis E., Chinou I.B., Mitakou S. (2001). Composition and antibacterial activity of the essential oils of five taxa of *Sideritis* from Greece. J. Agric. Food Chem..

[14] Couladis M., Chinou B., Tzakou O., Petrakis P.V. (2003). Composition and antimicrobial activity of the essential oil of *Hypericum rumeliacum* subsp. *apollinis* (Boiss. & Heldr.). Phytother. Res..

[15] Leite A.M., Lima E.O., Souza E.L., Diniz M.F.F.M., Trajano V.N., Medeiros I.A. (2007). Inhibitory effect of β-pinene, α-pinene and eugenol on the growth of potential infectious endocarditis causing Gram-positive bacteria. Braz. J. Pharm. Sci..

[16] Angioni A., Barra A., Russo A.T., Coroneo A., Dessiä S., Cabras P. (2003). Chemical composition of the essential oils of *Juniperus* from ripe and unripe berries and leaves and their antimicrobial activity. J. Agric. Food Chem..

[17] Koutsoudaki C., Krsek M., Rodger A. (2005). Chemical composition and antibacterial activity of the essential oil and the gum of *Pistacia lentiscus* var. chia. J. Agric. Food Chem..

[18] Zore G.B, Thakre A.D., Jadhav S., Karuppayil S.M. (2011). Terpenoids inhibit *Candida albicans* growth by affecting membrane integrity and arrest of cell cycle. Phytomedicine.

[19] Lewis K. (2001). Riddle of Biofilm Resistance. Antimicrob. Agents Chemother..

[20] Mowat E., Butcher J., Lang S., Williams C., Ramage G. (2007). Development of a simple model for studying the effects of antifungal agents on multicellular communities of *Aspergillus fumigatus*. J. Med. Microbiol..

[21] Clinical and Laboratory Standards Institute (CLSI) (2008). Methods for Dilution Antimicrobial Susceptibility Tests. Approved Standards, M27-A3, M38-A2, M11-A6 e M7-A4.

[22] Price M.F., Wilkinson I.D., Gentry L. (1982). Plate method for detection of phospholipase activity in *Candida albicans*. Sabouraudia.

[23] Schoofs A., Odds F.C., Colebunders R., Leven M., Goussens H. (1997). Use of specialized isolation media for recognition and identification of *Candida dubliniensis* isolates from HIV infected patients. Eur. J. Clin. Microbiol. Infect. Dis..

[24] Krom B.P., Cohen J.B., Feser G.E.M., Cihlar R.L. (2007). Optimized candidal biofilm microtiter assay. J. Microbiol. Methods.

[25] Mosmann T. (1983). Rapid colorimetric assay for cellular growth and survival: Application to proliferation and cytotoxicity assays. J. Immunol. Methods.

